# Retinoblastoma in Ceara: An epidemiological study in a Brazilian pediatric oncology referral center

**DOI:** 10.5935/0004-2749.2023-0265

**Published:** 2024-09-16

**Authors:** Samuel Montenegro Pereira, Rian Vilar Lima, Carlos Otavio de Arruda Bezerra Filho, Clara Memória Santos, Maria Carolina Rocha Muniz, Daiane Memória Ribeiro Ferreira, Juliana de Lucena Martins Ferreira

**Affiliations:** 1 Department of Medicine, Universidade de Fortaleza, Fortaleza, CE, Brazil; 2 Department of Medicine, Universidade Unichristus, Fortaleza, CE, Brazil; 3 Department of Pediatrics and Ophthalmology, Hospital Albert Sabin, Fortaleza, CE, Brazil

**Keywords:** Retinoblastoma, Retinal neoplasms, Epidemiology, Prevalence, Risk factors, Delayed diagnosis, Child

## Abstract

**Purpose:**

Although Brazil has a high prevalence of retinoblastoma, there is a lack of epidemiological data on the disease. Thus, in this study, we aimed to evaluate the epidemiological profile of patients diagnosed with retinoblastoma in the ophthalmology department of a pediatric tertiary referral hospital in Ceara, Brazil.

**Methods:**

A descriptive and cross-sectional study was conducted by retrospectively analyzing the clinical and socioeconomic data from the medical records of pediatric patients followed-up at the hospital between 2007 and 2021. Retinoblastoma was diagnosed on the basis of a fundoscopic or histopathologic examination.

**Results:**

The data of 105 patients were included in the study, and the mean patient age at the time of diagnosis was 1.7 years. Most of the patients were women (50.5%) and hailed from rural areas (57.4%), which was associated with a higher tumor stage. Of the 150 patients, 57.1% initially presented with leukocoria. Ocular hyperemia was associated with more advanced stages of retinoblastoma (p=0.004). Bilateral involvement was observed in 25.7% of the patients and at a significantly younger age (p=0.009). The presence of retinal detachment, vascularized lesions, and vitreous seeds significantly increased the likelihood of requiring enucleation.

**Discussion:**

This study presents an epidemiological description of retinoblastoma in Brazil, which highlights the significance of early detection. Delayed diagnosis is associated with a poorer visual prognosis and higher mortality rate, particularly in patients with unilateral disease. Risk factors for a more severe disease were retinal detachment, vascularized lesions, and vitreous seeds. The correlation between histopathological features and clinical outcomes was limited.

**Conclusion:**

Further studies are required to assess the influence of ocular hyperemia, fundoscopic assessment, and histopathologic findings on the prognosis of retinoblastoma. Moreover, it is critical to devise interventions to reduce the time-to-diagnosis in rural areas.

## INTRODUCTION

Retinoblastoma (Rb) is the most common intraocular neoplasm occurring in children worldwide, with an estimated incidence of 1 case in 17,000 live births^(1)^. It accounts for approximately 3% of all malignant tumors in childhood and is more frequent in those aged <5 years^(2^,^3)^. Tumor carcinogenesis is associated with a biallelic mutation of the Rb1 tumor suppressor gene, which is located on chromosome 13q14.2. The mutation undermines the action of the Rb1 tumor suppressor gene as a cell cycle control checkpoint^(2)^.

Rb exhibits autosomal dominant genetic inheritance in approximately 35% of the patients in whom a preexisting germline mutation already exists^(1^,^2)^. In these patients, there is an increased risk of development of additional primary neoplasms such as sarcomas and melanomas^(2)^. The sporadic nonhereditary Rb accounts for the remaining 65% of the cases^(1)^.

Overall, the diagnostic approach in Rb is based on clinical history and physical examination, which typically requires indirect fundoscopy^(4^,^5)^. Furthermore, patient survival and improved vision outcomes mainly depend on the early diagnosis and timely treatment of Rb^(4)^. In low-income countries, which exhibit the highest incidence rates, prognosis is mainly affected by delays in diagnosis and therapeutic approaches^(5)^.

Recognizing the disease epidemiology is crucial for the development of health interventions that are appropriate to local circumstances. Challenges in the health system may jeopardize the management of patients with Rb, especially with regard to resource distribution and geographic accessibility^(2^,^5)^. Data regarding disease epidemiology is significantly scarce in some countries with higher prevalence rates, including Brazil. Thus, in this study, we aimed to evaluate the epidemiologic profile of patients diagnosed with Rb in the ophthalmology department of a tertiary care pediatric referral hospital in Ceara, Brazil.

## METHODS

### Study design, location, and period

This cross-sectional retrospective study was based on data obtained via a review of medical records of patients visiting the ophthalmology department of a tertiary referral hospital for pediatric oncology in Ceara, Brazil, between 2007 and 2021.

### Inclusion and exclusion criteria

Female and male children, aged <18 years, with confirmed Rb and fundoscopic and imaging examination data were included in the study. If the patient had undergone enucleation, the histopathological diagnosis was considered the definitive diagnosis. Children were excluded if investigations demonstrated that the ocular tumor was secondary to another lesion.

### Study protocol

The following data were collected from medical records using a standard survey: sociodemographic data, clinical manifestations at presentation, referral data, imaging findings, fundoscopic findings, therapeutic strategies, and histopathologic findings. The therapeutic strategies available at our center include laser transpupillary thermotherapy (TTT), three-drug systemic chemotherapy, radiotherapy, and enucleation. The study was carried out according to the Strengthening the Reporting of Observational Studies in Epidemiology (STROBE) guidelines, which is recommended for conducting observational studies^(6)^.

### Data analysis

Sociodemographic data, presenting symptoms, clinical follow-up data, referral for specialist evaluation, treatment, and the highest stage of the more severely affected eye of each child were analyzed individually using descriptive statistics and Pearson’s chi-square test. The t-test was used for comparing means. In addition, treatment, fundoscopic findings, and histopathological findings (if the eye was enucleated) of each eye were assessed using descriptive statistics and the same tests as those used for the assessment of individual variables. A more detailed statistical analysis of the characteristics of patients who died during the study period was also performed.

### Ethical aspects

The study was approved by the hospital’s Research Ethics Committee (No: 3.424.628. The need for informed consent was waived due to the retrospective nature of the study (or the use of deidentified data).

## RESULTS

The medical records review revealed that 107 patients were diagnosed with Rb on the basis of fundoscopic examination. Of the 107 patients, 95 (88.8%) had undergone enucleation of at least one eye. These were subjected to histopathological examination, which revealed only two false-positive diagnoses. Finally, 105 patients (132 eyes) were included in the statistical analysis.

The mean age of the patients at the time of diagnosis was 1.7 years. The patients were predominantly female (50.5%) and mostly hailed from rural zones (57.4%). Leukocoria was the first symptom in a majority (57.1%) of the patients. Approximately 88.9% of the patients were referred by ophthalmologists, and 25.7% were diagnosed with bilateral disease. The median age at diagnosis of the patients with bilateral disease was significantly lower than those with unilateral disease (0.74 vs. 2.01 years; p=0.009). At the time of diagnosis or during the preoperative period, 90.5% of the patients underwent computed tomography (CT). Approximately 18.1% of these imaging studies demonstrated optic nerve invasion and damage (Table 1).

**Table 1:** Demographic data and clinical features of the study participants

Variable	Characteristic	Frequency	Percentage
Sex	Male	52	49.50
	Female	53	50.50
Origin	Capital	43	42,60
	Rural region	58	57,40
Referral	Ophthalmologist	64	88.90
	Pediatrician	4	5.60
	Emergency room	3	4.20
	Neurosurgeon	1	1.40
Laterality	Unilateral	78	74.30
	Bilateral	27	25.70
Imaging modality used	Computerized tomography	86	90.50
	Magnetic resonance imaging	13	13.70
	Ultrasound	14	14.70
Optic nerve invasion	Present	17	18.10
	Absent	77	81.90
Presenting symptom	Leukocoria	60	57.10
	Strabismus	18	17.10
	Hyperemia	9	8.60
	Eye pain	8	7.60
	Proptosis	1	1.00

**Figure 1:**
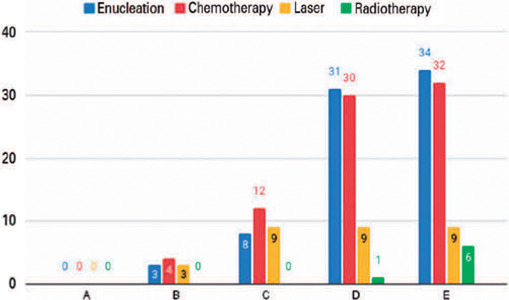
Distribution of treatment modality according to the tumor staging.

Based on the severity of the disease, 0%, 4.8%, 14.3%, 39.3%, and 41.7% of the patients were categorized into groups A, B, C, D, and E, respectively (Figure 1). Most of the patients underwent systemic chemotherapy (93.3%), followed by enucleation (90.5%), TTT (32.4%), and radiotherapy (7.6%). Five patients (4.8%) underwent bilateral enucleation. During the follow-up period, eight patients (7.6%) died.

The patient’s origin from a rural area was significantly associated with a higher Rb stage (p=0.32). Ocular hyperemia was also associated with a higher tumor stage (p=0.004), and ocular pain was marginally associated with the same staging feature (p=0.067).

The fundoscopic examination revealed the presence of retinal detachment, calcified lesions, vitreous seeds, and vascularized lesions in 16.2%, 9.2%, 8.1%, and 6.7% of the patients, respectively. In 16.1% of the patients, the tumor demonstrated extensive retinal involvement, which made visualization difficult or impossible. Table 2 shows the fundoscopy findings in relation to the outcome of the patients who did and did not undergo enucleation.

**Table 2:** Correlation between fundoscopy findings and patient outcome

		With enucleation	Without enucleation	p-value	Odds ratio	95% confidence interval
Retinal detachment	Present	33	1	<0.001	48.803	6.525-364.998
	Absent	71	105			
Vitreous seeds	Present	13	4	0.02	3.643	1.147-11.571
	Absent	91	102			
Vascularized lesion	Present	12	2	0.005	6.783	1.479-31.105
	Absent	92	104			
Calcified lesion	Present	12	8	0.325	1.598	0.625-4.085
	Absent	92	98			

Retinal detachment, vitreous seeds, and vascularized lesions were associated with significantly higher chances of requiring enucleation than calcified lesions. Moreover, retinal detachment, even if partial, constituted an almost absolute indication for enucleation. This was evidence by the 49-fold greater chance of requiring enucleation in the presence of retinal detachment.

The histopathological characteristics of the 104 enucleated eyes are included in table 3. There were no statistically significant relationships between the staging or fundoscopy findings and any of the histopathological features.

**Table 3:** Histopathological findings of the enucleated eyes

		Frequency	Percentage
Differentiation	Well differentiated	13	14.60
	Moderately differentiated	32	36.00
	Poorly differentiated	28	31.50
	Undifferentiated	16	18.00
Infiltration of the lamina cribrosa	Before	22	21.60
	In the region	9	8.80
	After	14	13.70
Choroidal infiltration	Focal	28	27.50
	Diffuse	22	21.60
Necrosis	-	30	28.80
Dystrophic calcification	-	12	11.50

Most of the patients who died during the follow-up period were male (62.5%), and they predominantly hailed from the capital (75%). The average age of these patients was 2.12 years, and ocular hyperemia was the main symptom. In 50% of the patients who died, visualization the retina was challenging due to the tumor extension. One patient had trilateral Rb, one exhibited iris invasion by the tumor, and another exhibited concomitant ocular cellulitis. The characteristics of these patients are summarized in table 4.

**Table 4:** Characteristics of the patients who died during the follow-up

Characteristics		Value
Age, mean		2.125 (1-4)
Sex, n (%)	Male	5 (62.5)
	Female	3 (37.5)
Origin, n (%)	Capital	6 (75)
	Rural region	2 (25)
Referral, n (%)	Ophthalmologist	4 (50)
Imaging modality used, n (%)	Tomography	4 (50)
	Tomography and ultrasound	1 (12.)
	Tomography and magnetic resonance	1 (12.5)
Optic nerve invasion, n (%)	Present	2 (25)
Clinical sign, n (%)	Redness	2 (25)
	Proptosis	1 (12.5)
	Convulsion	1 (12.5)
	Eye pain	1 (12.5)
Laterality, n (%)	Unilateral	8 (100)
	Bilateral	0 (0)
Fundoscopic findings, n (%)	Retina cannot be visualized	4 (50)
	Vascularized lesion	1 (12.5)
	Iris infiltration	1 (12.5)
Tumor stage, n (%)	C	1 (12.5%)
	D	1 (12.5)
	E	3(37.5)
Treatment n, (%)	Chemotherapy	7 (87.5)
	Enucleation	6 (75)
	Laser (TTT)	1 (12.5)

## DISCUSSION

Our study provides a comprehensive epidemiological analysis of a cohort of patients diagnosed with Rb in a region that has received limited scientific attention. We emphasized the clinical, fundoscopic, and histopathologic aspects of the tumor and demonstrated a correlation between them and disease prognosis. Furthermore, although the study was conducted at a center without access to the most current treatments and the frequency of late diagnoses was high, the mortality rate was low.

Rb is a prevalent childhood disease of the eye, with approximately 8,000 new cases being diagnosed annually worldwide^(1)^. It is the leading cause of death worldwide due to ocular cancer and the most common ocular neoplasia in children^(7)^. Rb typically develops in early childhood and can have severe negative consequences if not properly treated. The late diagnosis of Rb accounts for >70% of the global mortality rate^(5^,^8)^.

Epidemiologic data on Rb are limited due to its rarity and potential challenges in making a diagnosis, particularly in low-income countries such as Africa, Asia and Latin America, where >80% of the patients with RB reside^(7)^. Recent population-based studies have demonstrated high incidence rates of the disease in Brazil, with approximately 400 new cases diagnosed annually^(9^,^10^,^11)^.

A recent population-based study analyzed official Brazilian databases and found that the northeast region had the highest incidence of Rb^(12)^. This data confirms the trend of a disproportionate incidence of Rb in poorer regions^(13)^, which may be underestimated^(12)^. Although the Rb-associated mortality rate in Brazil remains higher than the world averages^(5^,^7^,^13)^, it has not increased at the same rate as the incidence levels, indicating a tendency of Rb to persist in most Brazilian regions^(12^,^14)^. Recent advancements in treatment modalities and efforts to disseminate evidence-based screening programs have likely contributed to the improvement in mortality rates^(14^,^15^,^16)^.

Rb is usually diagnosed before the age of 4 years in >90% of the cases^(12)^. In upper-middle-income countries such as Brazil, the average age at the time of diagnosis is approximately 20 months^(5)^. This data is consistent with that of our study, in which the average age at the time of diagnosis was 20.4 months. In high-income countries, the average age of at the time of diagnosis is approximately 14 months^(5^,^13)^.

Complementary imaging tests are generally used to aid in the diagnosis of diseases. Of these, CT is the most sensitive and accurate method for detecting intratumoral calcium deposition^(5^,^11)^. However, routine CT scans in children should be avoided due to the potential risk of radiation exposure and increased risk of a second neoplasm, particularly in patients with germinal Rb^(17)^. Nevertheless, due to limited access to alternative diagnostic methods in certain geographic locations, CT use remains prevalent in many regions of Brazil, including Ceara^(18)^.

In countries with patients of lower socioeconomic status, approximately 40% of the patients with Rb die during the medium- or long-term follow-up due to metastatic dissemination resulting from a late diagnosis^(11)^. This aspect may be even more pronounced in rural regions within developing countries, as evidenced in the present study and in other studies conducted in Latin American countries^(19)^. In contrast, the prognosis of patients in developed countries has improved in recent years, with high rates of disease-free survival being achieved. This improvement in prognosis is mainly attributed to the formation of specialized referral centers and the organization of orderly screening programs^(2^,^5)^.

The most common clinical manifestation of Rb is reportedly leukocoria (60%), which is characterized by an altered red reflex that is often first noticed by family members^(1)^. Similarly, the prevalence of leukocoria in our study was 57.1%. Strabismus is the second most frequent presentation of RB, and it is usually associated with macular involvement^(1^,^8)^. Other early clinical manifestations of Rb include decreased visual acuity and chronic uveitis^(4)^. In advanced cases, proptosis, congestive intraocular signs, and neovascular glaucoma may be present^(1^,^4^,^8)^. Our study revealed an association between ocular hyperemia and a higher Rb stage, which may be attributed to the congestion caused by large tumors.

Approximately two-third of the patients with Rb have unilateral involvement, and bilateral involvement is generally identified in younger children^(3^,^8)^. Trilateral Rb is a rare condition (<10%) in which bilateral eye involvement is associated with an asynchronous intracranial neoplasm^(1)^. Rb causes metastatic invasion in approximately 10% of the cases, and it is typically associated with deep choroidal spread, which causes death in most patients within 6 months of diagnosis^(20)^.

The global approach to patients with Rb is timely tumor diagnosis, appropriate treatment strategies, and clinical follow-up to identify possible recurrences^(1)^. Currently, the treatment modalities available are aimed at preserving the patient’s life, which entails prevention of extraocular invasion^(1^,^21)^. Depending on the location and extent of tumor involvement, enucleation, different chemotherapy combinations, laser therapy, cryotherapy, or radiation may be considered^(2)^.

Although delayed diagnosis and older age are the primary risk factors for metastatic disease and subsequent Rb-related death^(22)^, global studies have demonstrated that the poor prognosis in lower-income countries is not primarily due to the unavailability of treatment, but due to poor screening^(5^,^7^,^13)^. Our findings of tendency for a delayed diagnosis and higher mortality in unilateral Rb are consistent with the findings of previous studies^(16^,^23)^. Delayed diagnosis is mostly attributable to the challenges in detection.

The use of chemotherapy, TTT, radiation, and enucleation in our study reflect the individualized treatment trend commonly reported in low-resource centers^(23^,^24)^. However, the mortality rate in our study (7.6%) was comparable to that of high-resource centers in other regions of Brazil^(24)^. This indicates that the mortality rate can be significantly reduced even without more advanced treatment modalities.

The indication for enucleation remains controversial, particularly among patients with group D and E Rb. These cases have demonstrated modest ocular survival rates of approximately 19%, even after multimodal treatments in large centers^(25)^. Thus, we aimed to identify the fundoscopic findings that were most relevant and had the greatest impact on the decision to enucleate. We found that retinal detachment, vascularized tumor, and vitreous seeding were the most relevant factors. The definition of Rb severity is yet to be standardized between different centers^(26)^.

In developing countries, where metastatic disease and treatment dropout rates are high, strategies for salvaging the ocular globe are crucial.^(4)^. Furthermore, in these geographic regions, prompt diagnosis is essential to ensure better therapeutic outcomes^(27)^. Therefore, the identification of high-risk histopathological characteristics, particularly with respect to the extent of ocular involvement, is crucial as it may indicate an increased risk of metastasis^(27^,^28)^.

In our analysis, no histopathologic characteristics appeared to be related to the clinical features, fundoscopic findings, or prognosis. This finding is consistent with that of a previous study that also found no significant correlations between clinical presentation and histopathological characteristics^(28)^. Nevertheless, identifying typical high-risk features, as defined by the International Retinoblastoma Staging Working Group^(29)^, has traditionally been useful in guiding therapy after enucleation, particularly in cases of suspected or confirmed metastasis^(30)^. Therefore, the absence of a statistical correlation in this observation may be explained by the fact that patients with risk factors are already being closely monitored and treated.

This study has several limitations. This was a cross-sectional study, and detailed data regarding treatment, metastasis, and relapse rates could not be recorded. The epidemiological description presented in this study makes a significant contribution to literature, and the fundoscopic findings of this study may aid in the development of a standardized definition of high-risk Rb and an appropriate protocol for enucleation.

Our study findings demonstrate the epidemiological trends of Rb in Brazil and draw attention to the need for reduction in the age at the time of diagnosis of patients from rural areas. Our study findings also highlight some potential indicators of poor prognosis and the need for enucleation that should be investigated in further prospective studies. In future studies symptoms and fundoscopic findings should be evaluated to determine a more standard treatment approach, particularly regarding enucleation.
